# Near-Infrared Photoimmunotherapy Using a Protein Mimetic for EGFR-Positive Salivary Gland Cancer

**DOI:** 10.3390/ijms25063233

**Published:** 2024-03-12

**Authors:** Haruka Yamaguchi, Takamasa Suzuki, Yasuo Okada, Junya Ono, Hiroto Sano, Akiko Banba, Hideyuki Sakata, Akihiro Ishikawa, Takao Morita

**Affiliations:** 1 Department of Biochemistry, School of Life Dentistry at Niigata, The Nippon Dental University, Niigata 951-8580, Japan; moritat@ngt.ndu.ac.jp; 2 Faculty of Engineering, Niigata University, Niigata 950-2181, Japan; takamasa@eng.niigata-u.ac.jp; 3 Department of Pathology, School of Life Dentistry at Niigata, The Nippon Dental University, Niigata 951-8580, Japan; yokada@ngt.ndu.ac.jp (Y.O.); johno@ngt.ndu.ac.jp (J.O.); h-sano@ngt.ndu.ac.jp (H.S.); 4 Startup Incubation Center, Shimadzu Corporation, Kyoto 604-8511, Japan; nagata_a@shimadzu.co.jp (A.B.); sakata@shimadzu.co.jp (H.S.); ishikawa@shimadzu.co.jp (A.I.)

**Keywords:** NIR-PIT, Affibody, IR700Dye, EGFR, salivary gland cancer

## Abstract

Near-infrared photoimmunotherapy (NIR-PIT) is a novel cancer therapy based on a monoclonal antibody (mAb) conjugated to a photosensitizer (IR700Dye). The conjugate can be activated by near-infrared light irradiation, causing necrotic cell death with high selectivity. In this study, we investigated NIR-PIT using a small protein mimetic (6–7 kDa, Affibody) which has more rapid clearance and better tissue penetration than mAbs for epidermal growth factor receptor (EGFR)-positive salivary gland cancer (SGC). The level of EGFR expression was examined in vitro using immunocytochemistry and Western blotting. Cell viability was analyzed using the alamarBlue assay. In vivo, the volume of EGFR-positive tumors treated with NIR-PIT using the EGFR Affibody–IR700Dye conjugate was followed for 43 days. It was found that NIR-PIT using the EGFR Affibody–IR700Dye conjugate induced the selective destruction of EGFR-positive SGC cells and restricted the progression of EGFR-positive tumors. We expect that NIR-PIT using the EGFR Affibody–IR700Dye conjugate can efficiently treat EGFR-positive SGC and preserve normal salivary function.

## 1. Introduction

In 2011, Mitsunaga et al. reported a new type of cancer therapy using near-infrared light irradiation, called near-infrared photoimmunotherapy (NIR-PIT) [1]. Recently, it has been common to employ photodynamic therapy (PDT) in cancer treatment. PDT is a technology in which photosensitizer-mediated light irradiation produces reactive oxygen species (ROS) that kill cancer cells [2]. Although NIR-PIT uses a photosensitizer, its mechanism of action differs from that of conventional PDT. NIR-PIT is based on a monoclonal antibody (mAb) that is conjugated to a photosensitizer, IR700Dye. When the conjugate binds to the target receptor on the cancer cells, near-infrared light is used to activate the conjugate. Upon near-infrared light irradiation, IR700Dye undergoes photochemical ligand reactions that release hydrophilic side chains. This causes the remaining molecules to become hydrophobic. This chemical change leads to physicochemical changes within the IR700Dye-mAb conjugate. These events reduce cell membrane integrity due to damage to transmembrane target proteins. Because of osmotic pressure, the damaged cell membrane allows water to enter the cells, which eventually burst, causing necrotic cell death [3,4,5,6]. Currently, NIR-PIT targeting epidermal growth factor receptor (EGFR) with an mAb–IR700Dye conjugate is under global phase III clinical evaluation for the treatment of head and neck cancers (NCT03769506).

In this study, we investigated the effectiveness of NIR-PIT using a small protein mimetic, Affibody, instead of a monoclonal antibody for the treatment of EGFR-positive salivary gland cancer (SGC). Affibody is a small alpha-helical polypeptide ligand that is synthesized based on an immunoglobulin-binding region of staphylococcal protein A to recognize various molecules. The molecular weight of Affibody is just 6–7 kDa. Due to its small size, Affibody has more rapid clearance and better tissue penetration than mAb [7,8]. In addition, Affibody possesses biocompatibility as well as impressive chemical and thermal stability [9]. Clinical applications of Affibody have already been reported [10]. Therefore, we hypothesized that Affibody represents an appropriate therapeutic agent for NIR-PIT and suggest that it has the potential to be safely and quickly translated into clinical practice.

EGFR is expressed in approximately 40–65% of salivary gland cancer cases [11,12]. Therefore, NIR-PIT using the EGFR Affibody–IR700Dye conjugate for SGC may be a better cancer treatment option than conventional treatment approaches such as surgery, chemotherapy, and radiotherapy, which greatly affect the salivary secretion system and facial nerve that passes through the parotid gland [13]. We expect that it will expand the targeting scope of NIR-PIT for EGFR-positive SGC.

## 2. Results

### 2.1. Epidermal Growth Factor Receptor (EGFR) Expression

**Figure 1:**
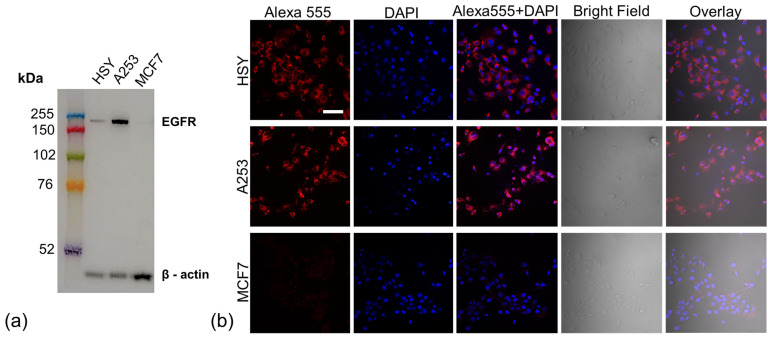
(**a**) Western blotting (WB) of epidermal growth factor receptor (EGFR) protein on salivary gland cancer cells (HSY, A253) and breast cancer cells (MCF7, negative control). A strong band with a molecular weight of 170 kDa, corresponding to EGFR protein, was observed in HSY cells and A253 cells. Measures of 30 µg of proteins, primary antibody (1:1000, Anti-Human EGFR Mab (Clone 102618)), and secondary antibody (1:5000, EasyBlot antimouse IgG (HRP), Gene Tex, Inc., Alton Pkwy, Irvine, CA, USA) were utilized in WB. (**b**) Immunocytochemistry of EGFR protein on salivary gland cancer cells exhibited stronger fluorescent signals than MCF7, in which EGFR expression was virtually undetected. Scale bar: 100 µm. In total, 1 × 10^5^ HSY cells, A253 cells, and MCF7 cells were seeded on coverslips at the bottoms of wells in a 24-well plate. Anti-EGFR antibody (1:1000, Human EGF R/ErbB1 MAb (Clone 102618)), the secondary antibody (1:1000, Anti-mouse IgG Alexa Fluor Plus 555 (Ex/Em = 553/568 nm)) and the mounting medium with DAPI (Ex/Em = 350/465 nm) were used.

Salivary gland cancer cells (HSY, A253), and breast cancer cells (MCF7, negative control) were investigated for epidermal growth factor receptor (EGFR) expression using Western blotting analysis and immunocytochemistry (ICC). In the Western blotting analysis, a strong band with a molecular weight of 170 kDa corresponding to the EGFR protein was observed in HSY and A253 cells. The band of HSY cells was weaker than that of A253 cells (Figure 1a). In ICC, HSY and A253 cells showed stronger fluorescent signals than MCF7 cells did, in which EGFR expression was virtually undetected (Figure 1b).

### 2.2. Fluorescence Image of the Cells Bound to the EGFR Affibody-IR700Dye Conjugate

**Figure 2:**
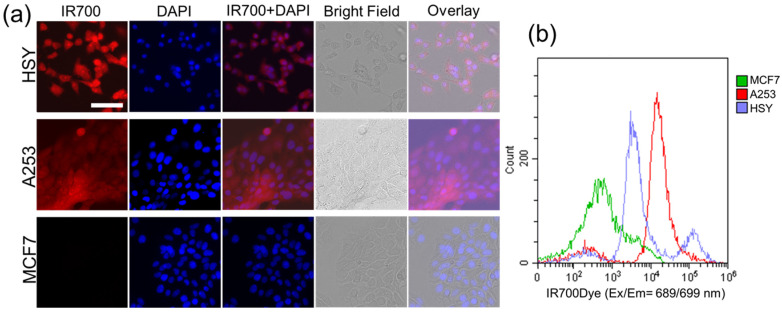
(**a**) Fluorescence image of the cells bound to EGFR Affibody–IR700Dye conjugate. EGFR-positive cells (A253, HSY) exhibited stronger fluorescent signals of IR700Dye than EGFR-negative cells (MCF7). Scale bar: 100 µm. In total, 1 × 10^5^ HSY cells, A253 cells, and MCF7 cells were seeded on the glass bottoms of wells in a 24-well plate. The EGFR Affibody–IR700Dye conjugate (1 µM) was added to the medium. (**b**) Flow cytometry after labeling of the EGFR Affibody–IR700Dye conjugate to EGFR receptors. IR700Dye showed a strong fluorescence intensity in HSY cells and A253 cells compared to MCF7 cells as control. It revealed the specific binding of EGF receptors. In total, 1 × 10^6^ cells were added to 1 µM of the EGFR Affibody–IR700Dye conjugate. One thousand events were counted.

HSY and A253 cells exhibited stronger fluorescent signals of IR700Dye than MCF7 cells did, as demonstrated by the addition of the EGFR Affibody–IR700Dye conjugate. The fluorescence images showed similar fluorescence intensity to the image obtained using ICC (Figure 2a). Flow cytometry after labeling of the EGFR Affibody–IR700Dye conjugate showed a strong fluorescence intensity of IR700Dye in HSY cells and A253 cells compared to MCF7 cells as a control. It revealed specific binding of EGF receptors (Figure 2b).

### 2.3. Effect of NIR-PIT Using the EGFR Affibody-IR700Dye Conjugate on the Cells

**Figure 3:**
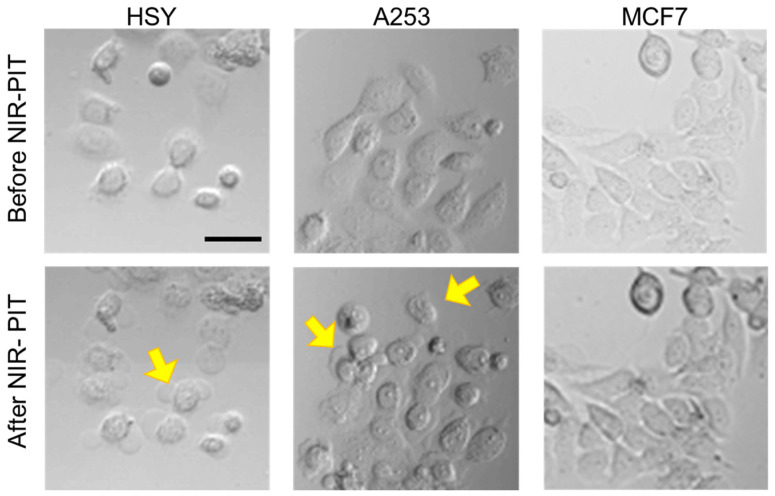
Cell images before and after NIR-PIT using the EGFR Affibody–IR700Dye conjugate. The images showed that EGFR-positive cells (A253, HSY) displayed morphological evidence of cellular bursting and bleb formation (yellow arrows). Scale bar: 50 µm. The cells were seeded at 1 × 10^4^/well in 96-well culture plates, followed by incubation with the EGFR Affibody–IR700Dye conjugate (0.5 µM).

**Figure 4:**
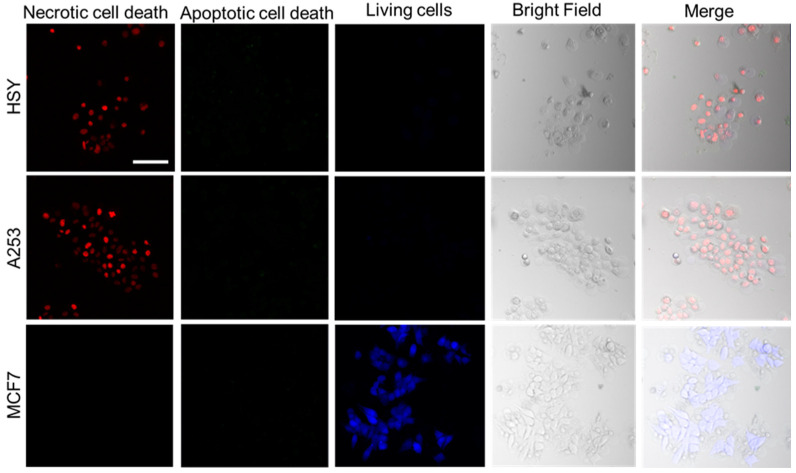
The Apoptosis/Necrosis Assay Kit stains living cells blue (Ex/Em = 405/450 nm), apoptotic dead cells green (Ex/Em = 490/525 nm), and necrotic dead cells red (Ex/Em = 546/647 nm). EGFR-positive cells (A253, HSY) exposed to NIR-PIT using the EGFR Affibody–IR700Dye conjugate stained red, whereas EGFR-negative cells (MCF7) stained blue. Scale bar: 100 µm. The cells were seeded at 1 × 10^4^/well in 96-well culture plates, followed by incubation with the EGFR Affibody–IR700Dye conjugate (0.5 µM).

The images of the cancer cells showed that HSY and A253 cells exposed to NIR-PIT using the EGFR Affibody–IR700Dye conjugate displayed morphological evidence of cellular bursting and bleb formation, whereas the morphology of MCF7 cells remained unchanged (Figure 3). Furthermore, as shown in Figure 4, in the apoptosis/necrosis assay, HSY and A253 cells exposed to NIR-PIT using the EGFR Affibody–IR700Dye conjugate showed red signals (necrotic cell death), while MCF7 cells were stained blue (living cells).

### 2.4. Cell Viability after Near-Infrared Photoimmunotherapy (NIR-PIT)

**Figure 5:**
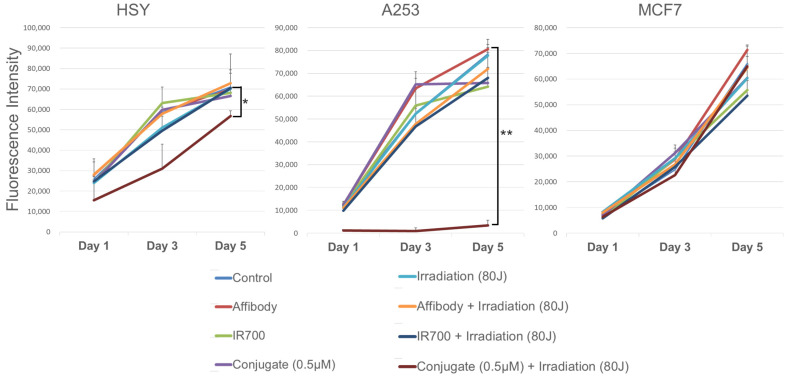
alamarBlue assay after NIR-PIT using the EGFR Affibody–IR700Dye conjugate. When the cell viability was measured over an extended period of 5 days, only the EGFR-positive cells (A253, HSY) exposed to near-infrared light irradiation and the EGFR Affibody–IR700Dye conjugate were statistically low. Data are presented as means ± SD (*n* = 6; * *p* < 0.05; ** *p* < 0.01, one-way ANOVA with Tukey–Kramer post hoc tests). The cells were seeded at 1 × 10^4^/well in 96-well plates and allowed to grow for 24 h, followed by incubation with Affibody only, IR700Dye only, or HER2 Affibody–IR700Dye conjugate (0–0.5 µM).

The alamarBlue assay showed cell viability as a fluorescence intensity. As shown in Figure 5, HSY and A253 cells incubated with the EGFR Affibody–IR700Dye conjugate and irradiated with NIR light (80 J/cm^2^) maintained lower cell viability compared to the other samples, even five days after irradiation. On the other hand, all samples of MCF7 cells increased rapidly, including the cells that were exposed to the conjugates (0.5 µM each) and irradiated with NIR light (80 J/cm^2^). The results of representative experiments, which used six wells per sample and were repeated more than three times, are presented as the mean ± SD (** *p* < 0.01 vs. non-treatment control).

### 2.5. In Vivo Fluorescence Images and Immunohistochemistry

**Figure 6:**
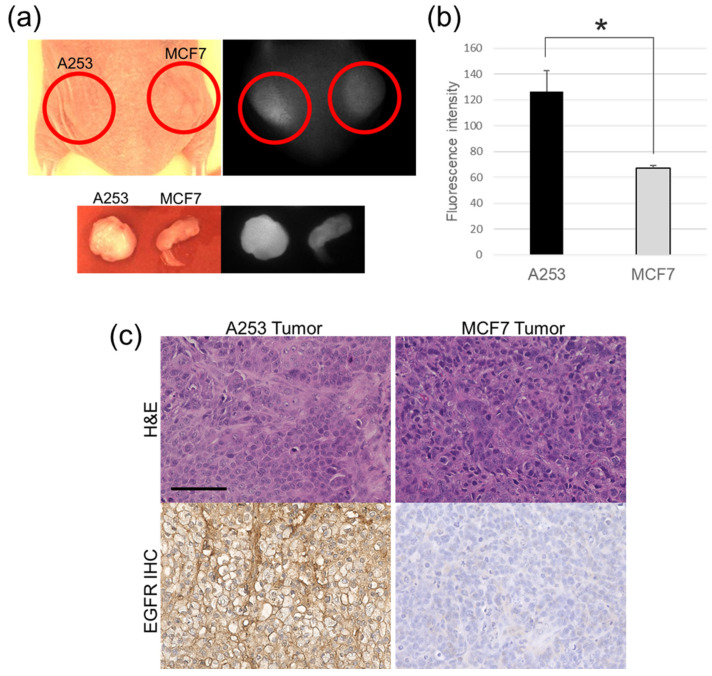
(**a**) In vivo imaging of A253 tumor xenograft-bearing mice with the EGFR Affibody–IR700Dye conjugate. The image shows high-intensity IR700Dye fluorescence in EGFR-positive A253 tumor compared to EGFR-negative MCF7 tumor. To establish each tumor, 1 × 10^7^ cells suspended in 150 µL of a 1:1 mixture of Matrigel (Becton Dickinson) and PBS were subcutaneously injected into the dorsum. Two weeks later, the EGFR Affibody–IR700Dye conjugate (100 µL) was injected into the tail vein. (**b**) The fluorescence intensities of IR700Dye showed a significant difference between A253 tumors and MCF7 tumors (*n* = 3, * *p* < 0.05, Student’s *t*-test). Data are presented as means ± SD. (**c**) The immunohistochemistry of EGFR protein from the engrafted tumors revealed stronger positive EGFR staining on the cell membrane of the A253 tumor than the MCF7 tumor (negative control). Scale bar: 100 µm. Serial sections of 3 μm in thickness were prepared. The sections were incubated with the primary antibody (1:200, Anti-Human EGFR Mab (Clone 102618) R&D SYSTEMS, Minneapolis, MN, USA) and secondary antibodies (Histofine Simple Stain MAXPO MULTI; Nichirei Bioscience Inc., Tokyo, Japan).

EGFR-positive A253 tumors exhibited a strong fluorescence intensity of IR700Dye compared to EGFR-negative MCF7 tumors, as shown in in vivo and ex vivo images (Figure 6a). In addition, analysis using Student’s *t*-test (A253; *n* = 3, MCF7; *n* = 3) indicated that there was a significant difference in fluorescence intensity between the A253 tumors and the MCF7 tumors (* *p* < 0.05) (Figure 6b).

**Figure 7:**
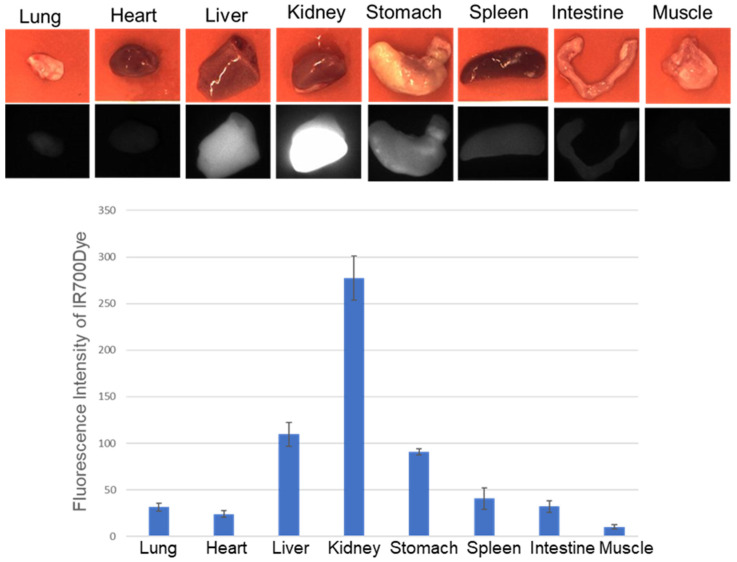
Fluorescence intensity from the individual organs of the mice injected with 100 µL of the EGFR Affibody–IR700Dye conjugate. Data are presented as means ± SD (*n* = 3). The liver and kidney showed strong fluorescence intensity of IR700Dye.

EGFR immunohistochemistry of engrafted tumors revealed stronger EGFR-positive stains in the A253 tumor than in the MCF7 tumor (Figure 6c). Ex vivo, the fluorescence intensities from the organs showed the strong fluorescence intensity of IR700Dye in the liver and kidney (Figure 7).

### 2.6. Tumor Volume after NIR-PIT

**Figure 8:**
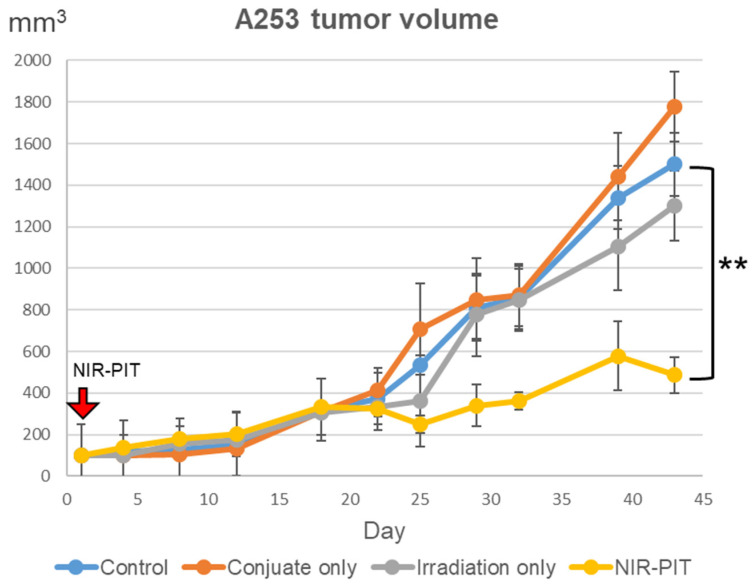
Tumor volume after NIR-PIT using the EGFR Affibody–IR700Dye conjugate. It was significantly restricted in A253 tumors treated with NIR-PIT compared to untreated control. We observed no significant therapeutic effect in any other groups with either injection of the EGFR Affibody–IR700Dye conjugate only or near-infrared light irradiation only. Data are presented as means ± SD (*n* = 6; ** *p* < 0.01, one-way ANOVA with Tukey–Kramer post hoc tests). The A253 mouse model was established by injecting 1 × 10^7^ cells suspended in 150 µL of a 1:1 mixture of Matrigel (Becton Dickinson) and PBS subcutaneously into the dorsum. The amount of the EGFR Affibody–IR700Dye conjugate injected into the tail vein was 100 µL. The irradiated near-infrared light was 150 J/cm^2^.

The results presented in Figure 8 show that the volume of the tumors in the group treated with NIR-PIT using the EGFR Affibody–IR700Dye conjugate displayed efficient suppression of A253 tumors over 40 days. In contrast, the tumors in the other groups showed a rapid increase that was similar to that observed in the control group (non-treatment). The results of representative experiments are presented as the mean ± SD (A253: *n* = 6, MCF7: *n* = 7, ** *p* < 0.01 vs. non-treatment control).

## 3. Discussion

Immunocytochemistry (ICC) and Western blot analysis demonstrated the positive expression of the EGFR protein in HSY and A253 cells compared to that in MCF7 cells (Figure 1). These results are in agreement with previous reports [14,15,16]. Fluorescence imaging of the EGFR-positive cells (HSY, A253) applied to the EGFR Affibody–IR700Dye conjugate showed a similar fluorescence intensity of IR700Dye to ICC (Figure 1b and Figure 2a), suggesting that the EGFR Affibody–IR700Dye conjugate bound to the EGFR protein on EGFR-positive cells was highly specific.

The results of the cell morphological changes and the Apoptosis/Necrosis Assay indicated that EGFR-positive cells treated with NIR-PIT using the EGFR Affibody–IR700Dye conjugate underwent selective necrotic cell death without any damage to EGFR-negative cells (Figure 3 and Figure 4). According to Sato et al., NIR-PIT using mAb induces physical changes in the conjugate that is bound to the surface of the target cells, exerting physical stress within the cellular membrane and leading to an overall increase in transmembrane water flow that eventually leads to cell bursting and necrotic cell death [6]. It is speculated that NIR-PIT using the EGFR Affibody–IR700Dye conjugate causes a similar amount of physical stress against the cell membrane of targeted cells as the current NIR-PIT using mAb. As shown in Figure 5, although the survival of A253 cells was maintained at a low level even five days after NIR-PIT using the EGFR Affibody–IR700Dye conjugate, the cell survival in HSY cells was approximately 50–80% compared to that of the control cells. This was because the expression level of EGFR in HSY cells was lower than that of A253 cells, and the effect of NIR-PIT using the EGFR Affibody–IR700Dye conjugate must have been weaker in the HSY cells than in the A253 cells. The results of our previous study indicated that the effect of NIR-PIT is well correlated with the level of targeted protein expression in cells [17]. Therefore, HSY cells may require higher near-infrared light doses or higher concentrations of the conjugate to ensure complete cell death [18,19,20]. Alternatively, to improve the efficiency of NIR-PIT for HSY cells, NIR-PIT using a cocktail of antibody conjugates or combination therapy using both full antibody and Affibody would be more effective [21,22,23,24].

As indicated in both the in vivo and ex vivo images, the EGFR Affibody–IR700Dye conjugate clearly delineated the A253 tumor compared to the MCF7 tumor, suggesting that the EGFR Affibody–IR700Dye conjugate bound to the EGFR protein and imaged an EGFR-positive tumor (Figure 6a,b). In the ex vivo study, the fluorescence images showed the strong fluorescence intensity of the EGFR Affibody–IR700Dye conjugate not only in the tumor but also in the liver and kidney (Figure 6a,b and Figure 7). This is due to the fact that small proteins tend to be abundantly deposited in the kidney, which may be associated with the renal elimination and reabsorption of the Affibody molecules [25,26,27,28]. Despite these tendencies, we expect that NIR-PIT using the EGFR Affibody–IR700Dye conjugate is fully applicable for clinical use because Affibody and IR700Dye are already used clinically [29,30].

The finding that EGFR-positive salivary gland cancer can be effectively treated with NIR-PIT using the EGFR Affibody–IR700Dye conjugate may extend the therapeutic prospect of salivary gland cancer treatment. Approximately 40–70% of salivary gland cancers overexpress EGFR, and EGFR overexpression in recurrent or metastatic SGC was detected in 77.8% of cases [11,12,31,32]. Moreover, EGFR expression is associated with aggressive behaviors such as nerve invasion and impaired saliva production [33,34,35]. Therefore, NIR-PIT using the EGFR Affibody–IR700Dye conjugate is highly effective. In general, cancer treatment (surgery, radiotherapy, and chemotherapy) often causes severe side effects that have a negative impact on the quality of life [36,37]. The results of this study showed that utilizing NIR-PIT with the EGFR Affibody–IR700Dye conjugate may be an efficient strategy because Affibody has more rapid clearance and better tissue penetration than mAb [7,8]. When NIR-PIT using the EGFR Affibody–IR700Dye conjugate is considered for EGFR-positive SGC, near-infrared light can be irradiated directly using an optical fiber diffuser inserted into the tumor [38,39]. NIR-PIT using the EGFR Affibody–IR700Dye conjugate can preserve the function of the salivary gland as much as possible. Moreover, when treating cancer of the parotid gland, which the facial nerve passes through, NIR-PIT using the EGFR Affibody–IR700Dye conjugate can remove cancer cells as far into the gland as possible while preserving normal nerve cells.

Positive expression of EGFR has already been reported for oral squamous cell carcinoma, representing the most common type of malignancy in the oral cavity and esophagus [40,41]. Therefore, the EGFR Affibody–IR700Dye conjugate holds therapeutic potential for targeting these tumors as well.

This is the first ever report of NIR-PIT using the EGFR Affibody–IR700Dye conjugate for salivary gland cancer. Burley T.A. et al. also reported the effectiveness of NIR-PIT using Affibody for glioblastoma and breast cancer [42,43]. We believe that it can be applied to other types of systemic cancer as long as the targeted receptor is in the cell membrane.

This study clearly demonstrated that NIR-PIT using the EGFR Affibody–IR700Dye conjugate represents a new treatment strategy for EGFR-positive salivary gland cancer that is less invasive and improves the quality of life. Although further studies are required to examine the full therapeutic potential of this approach, NIR-PIT using the EGFR Affibody–IR700Dye conjugate is expected to become one of the most effective treatments for salivary gland cancer.

## 4. Materials and Methods

### 4.1. Cell Culture

The human parotid gland cancer cell line (EGFR+) HSY-EA1 (HSY) was generously gifted from Tokushima University, Japan [44]. The human submandibular gland cancer cell line (EGFR+) A253, which is derived from the acinar-intercalated duct region and the human breast cancer cell line (EGFR-) MCF7 were obtained from the American Type Culture Collection (ATCC^®^, Manassas, VA, USA). HSY and MCF7 cell lines were cultured in Dulbecco’s Modified Eagle Medium (DMEM, GIBCO^®^, Life Technologies, Carlsbad, CA, USA) supplemented with 10% fetal bovine serum and 1% penicillin–streptomycin. The A253 cell line was cultured in McCoy’s 5A medium (GIBCO^®^, Life Technologies, Carlsbad, CA, USA) supplemented with 10% fetal bovine serum (FBS; GIBCO^®^, Life Technologies, Carlsbad, CA, USA) and 1% penicillin–streptomycin (Invitrogen, Life Technologies, Carlsbad, CA, USA). All cell lines were maintained in a humidified environment containing 5% CO_2_ at 37 °C. The medium was changed every other day.

### 4.2. EGFR Affibody–IR700Dye Conjugate

EGFR Affibody (Affibody AB, Solna, Sweden) was dissolved in phosphate-buffered saline (PBS) to a final concentration of 1 mg/mL and dithiothreitol (DTT) was added to a final concentration of 20 mM at >pH 7.5. It was then incubated at room temperature for 2 h. To remove excess DTT from the conjugate, the EGFR Affibody was passed through a NAP5 column (GE Healthcare, Chicago, IL, USA). After that, the EGFR Affibody was incubated with a five-fold molar excess of IRDye700DX–maleimide (MW: 1979.23, LI-COR Biosciences, Lincoln, NE, USA) for 2 h at 37 °C. After conjugation, the solution was applied to protein desalting spin columns (Thermo Fisher Scientific, Waltham, MA, USA) and centrifuged at 1500× *g* for 2 min to purify the sample.

### 4.3. In Vitro Near-Infrared Photoimmunotherapy (NIR-PIT) Illuminator

**Figure 9:**
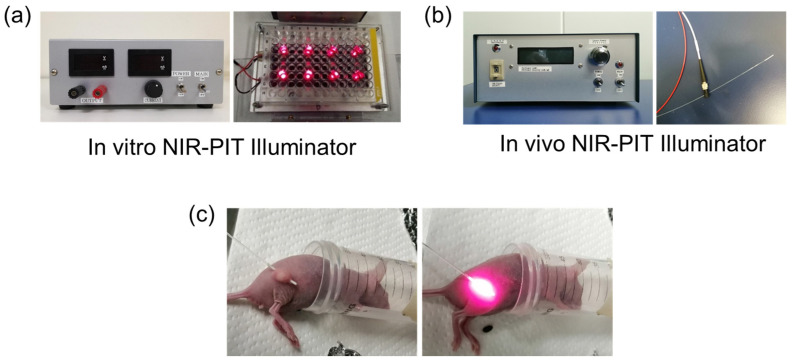
(**a**) The in vitro Near-Infrared Photoimmunotherapy (NIR-PIT) Illuminator is constructed of eight light-emitting diodes (LED: SMBB690D-1100-02 8, EPITEX, Inc., Kyoto, Japan), whose peak wavelength of emission is 690 nm. The power density of the LEDs is controllable from 0 to 244.86 mW/cm^2^ (0–600 mA). (**b**) The in vivo NIR-PIT Illuminator is a fiber-emitting laser irradiation system that is constructed of a laser diode (LD) receptacle module with an SMA connector and an LD. A cylindrical light diffuser (MODEL RD-ML-AS10) that allows the light source to insert into tumors can be connected. (**c**) A cylindrical light diffuser is inserted into the xenografted tumor, and the near-infrared light (150 J/cm^2^) is irradiated.

Our own designed NIR-PIT illuminator (Figure 9a) is constructed of eight light-emitting diodes (LED: SMBB690D-1100-02 8, EPITEX, Inc., Kyoto, Japan), whose peak wavelength of emission is 690 nm. The power density of the LEDs is controllable from 0 to 244.86 mW/cm^2^ (0–600 mA). In this study, the power density was set to 200 mW/cm^2^ at 500 mA. The power density passing through the bottom of the well was measured using an optical power meter, which was made by combining a photo diode detector (PH100-Si-HA, Gentec Electro-Optics, Inc., St-Jean-Baptiste, Quebec City, QC, Canada) and a touchscreen display device (MAESTRO, Gentec Electro-Optics, Inc., Quebec City, QC, Canada).

### 4.4. In Vivo Near-Infrared Photoimmunotherapy (NIR-PIT) Illuminator

The in vivo NIR-PIT Illuminator (Figure 9b) is a fiber-emitting laser irradiation system that is constructed with a laser diode (LD) receptacle module with an SMA connector assembled by Precise Gauges Co., Ltd., Shizuoka, Japan and an LD (HL6738MG, 690 nm, 35 mW, Ushio Inc., Tokyo, Japan). A cylindrical light diffuser (MODEL RD-ML-AS10) that allows the light source to insert into tumors can be connected (Figure 9b,c).

### 4.5. Immunocytochemistry (ICC)

In total, 1 × 10^5^ EGFR-positive salivary gland cancer cells (A253, HSY) and EGFR negative breast cancer cells (MCF7) were seeded on coverslips at the bottoms of wells in a 24-well plate. The cells were fixed in 4% paraformaldehyde for 10 min and washed with phosphate-buffered saline (PBS) twice, and non-specific sites were blocked with 3% bovine serum albumin (BSA) in PBS for 30 min at room temperature. Then, the cells were incubated with anti-EGFR antibody (Human EGF R/ErbB1 MAb (Clone 102618) (Mouse) R&D Systems, Inc., Lake Bluff, IL, USA) overnight at 4 °C, followed by incubation with the appropriate secondary antibody (1:1000, Anti-mouse IgG Alexa Fluor Plus 555, Thermo Fisher Scientific, Waltham, MA, USA) for 1 h at room temperature. After washing with PBS twice, the mounting medium with DAPI (Vector Laboratories, Newark, CA, USA) was added to the cover slips prior to imaging using a fluorescence microscope (LSM 700 confocal, ZEISS, Oberkochen, Germany).

### 4.6. Fluoresce Intensity of EGFR Affibody–IR700Dye Conjugate Staining

For fluorescence imaging, EGFR-positive salivary gland cancer cells (A253, HSY) and EGFR-negative breast cancer cells (MCF-7) were seeded on the bottoms of wells in a glass bottom 24-well plate. To test the specificity of the conjugate binding, the EGFR Affibody–IR700Dye conjugate (1 µM) was added to the medium, and the cells were incubated for 30 min at 37 °C. After washing the cells with PBS, they were examined using a fluorescence microscope (LSM confocal, ZEISS, Oberkochen, Germany).

For flow cytometry analysis, after detaching the cells with trypsin/ethylenediaminetetraacetic acid (EDTA), to a 1-milliliter cell suspension including 1 × 10^6^ cells, EGFR Affibody-IR700Dye conjugate (1 µM) was added. The cell suspension was incubated for 30 min at 37 °C, washed with PBS, and then, the fluorescence intensity was examined using a flow cytometer (CytoFLEX, Beckman Coulter Life Sciences, Inc., Brea, CA, USA).

### 4.7. Western Blot Analysis

Modified RIPA buffer (Thermo Fisher Scientific, Waltham, MA, USA) containing a protease inhibitor tablet (cOmplete™, Mini Protease Inhibitor Cocktail, Roche, Mannheim, Germany) was added to the cells (A253, HSY, and MCF7) to extract protein. Equal amounts of proteins (30 µg) were subjected to SDS-PAGE. Then, the proteins were transferred to a polyvinylidene fluoride (PVDF) membrane. After blocking with 5% non-fat milk, the membrane was incubated with primary antibody (Anti-Human EGFR Mab (Clone 102618) R&D SYSTEMS, Minneapolis, MN, USA) at 4 °C overnight. Subsequently, the membrane was incubated with secondary antibodies (EasyBlot anti-mouse IgG (HRP), Gene Tex, Inc., Alton Pkwy, Irvine, CA, USA). The immunoreactive bands were visualized with chemiluminescence using ECL Western blotting detection reagent (ECL Prime Western Blotting Detection Reagent, GE Health Care AmershamTM, Chicago, IL, USA). Image Quant LAS-500 (GE Healthcare, Chicago, IL, USA) was used for visual assessment.

### 4.8. Cell Viability Assay

The viability of the cells was determined by measuring the fluorescence intensity using an alamarBlue assay kit (Thermo Fisher Scientific, Waltham, MA, USA). Briefly, cells were seeded at 1 × 10^4^/well in 96-well plates and allowed to grow for 24 h, followed by incubation with Affibody only, IR700Dye only, or HER2 Affibody–IR700Dye conjugate (0–0.5 µM) for 2 h at 37 °C. After washing the cells twice with PBS, near-infrared light (0–80 J/cm^2^) was irradiated from the bottom of the wells. The cells were then incubated with alamarBlue solution (10 µL/100 µL in medium) for 2 h, and the fluorescence intensity was measured using a microplate reader (Power Scan’MX, DS PHARMA BIOMEDICAL, Osaka, Japan). The cell viability was observed for five days after NIR-PIT.

### 4.9. Cell Images before and after Near-Infrared Light Irradiation

The cells were seeded at 1 × 10^4^/well in 96-well culture plates and allowed to grow for 24 h, followed by incubation with the EGFR Affibody–IR700Dye conjugate (0.5 µM) for 2 h at 37 °C. After washing the cells twice with PBS, near-infrared light (80 J/cm^2^) was irradiated from the bottom of the wells. The images of the cells were taken using a microscope (LSM confocal, ZEISS, Oberkochen, Germany) before and after near-infrared irradiation.

### 4.10. Cell Apoptosis/Necrosis Assay

The cells (HSY, A253, and MCF-7) were seeded at 1 × 10^4^/well in 96-well culture plates and allowed to grow for 24 h, followed by incubation with the EGFR Affibody–IR700Dye conjugate (0.5 µM) for 2 h at 37 °C. After washing the cells twice with PBS, near-infrared light (80 J/cm^2^) was irradiated from the bottom of the wells. Then, apoptosis or necrosis of the cells was determined using the Apoptosis/Necrosis Assay Kit (ab176749, Abcam, Cambridge, UK) as described in the manufacturer’s protocol. This kit simultaneously detects cell necrosis (red), apoptosis (green), and healthy cells (blue) with a flow cytometer or fluorescence microscope. The images of the cells were taken using a fluorescence microscope (LSM confocal, ZEISS, Oberkochen, Germany).

### 4.11. In Vivo Study

Four-week-old female athymic mice (BALB/cSlc-nu/nu, Japan SLC, Shizuoka, Japan, body weight 12–17 g) were used in the animal studies. The mice were acclimatized for a week and housed under specific-pathogen-free (SPF) conditions with a 12 h light/dark cycle in cages. They were given free access to feed (D10001, AIN-76A, Research Diet Inc., New Brunswick, NJ, USA) and sterilized water.

A253 cells that were most affected by NIR-PIT in vitro study were chosen for the in vivo NIR-PIT study. The A253 mouse model was established by injecting 1 × 10^7^ cells suspended in 150 µL of a 1:1 mixture of Matrigel (Becton Dickinson, Franklin Lakes, NJ, USA) and PBS subcutaneously into the dorsum. Two weeks later, the mice were randomly divided into four groups. Group 1 was treated with NIR-PIT, injected with the EGFR Affibody–IR700Dye conjugate, and irradiated with 690 nm near-infrared light; group 2 was treated with injection of the EGFR Affibody–IR700 conjugate without irradiation; group 3 was treated with near-infrared light irradiation only; and group 4 was a control, thus receiving no treatment. A measure of 100 µL of the EGFR Affibody–IR700Dye conjugate was injected into the tail vein. Twenty-four hours after the injection, the tumors of the mice in groups 1 and 3 had a light source inserted into them and received irradiation with 150 J/cm^2^ of near-infrared light. The sizes of the tumors in all groups were measured for 43 days.

For the imaging study, 1 × 10^7^ A253 cells suspended in 150 µL of a 1:1 mixture of Matrigel (Becton Dickinson, Franklin Lakes, NJ, USA) and PBS were subcutaneously injected into the left dorsum. Simultaneously, 1 × 10^7^ MCF7 cells suspended in 150 µL of a 1:1 mixture of Matrigel (Becton Dickinson) and PBS were subcutaneously injected into the right dorsum. Two weeks later, the EGFR Affibody-IR700Dye conjugate was injected into the tail vein. Twenty-four hours after the injection, fluorescence whole-body images of mice were acquired using a Luminous Quester NI (Shimadzu, Kyoto, Japan). Subsequently, their organs were removed, and fluorescence images of each organ were taken using Luminous Quester NI (Shimadzu, Kyoto, Japan). Subsequently, the fluorescence intensity of the organs and tumors was analyzed using Image J software 1.52p (National Institutes of Health, Bethesda, MD, USA).

### 4.12. Immunohistochemistry

The excised tumors (A253 tumors with MCF7 tumors as a negative control) were fixed in 4% paraformaldehyde solution overnight and embedded in a paraffin block. Serial sections of 3 μm in thickness were prepared. Non-specific binding was blocked using incubation in 3% BSA (Sigma Aldrich, Burlington, MA, USA) for 30 min at room temperature. Then, the sections were incubated with the primary antibody (1:200, Anti-Human EGFR Mab (Clone 102618) R&D SYSTEMS, Minneapolis, MN, USA) overnight at 4 °C. After washing with PBS, the sections were then incubated with secondary antibodies (Histofine Simple Stain MAXPO MULTI; Nichirei Bioscience Inc., Tokyo, Japan) for 30 min at room temperature. The color was developed using 3,3′-diaminobenzidine·4HCl (DAB Substrate Kit; Nichirei Bioscience Inc.). After nuclear staining with hematoxylin, the slides were observed under a light microscope (IX71, Olympus, Tokyo, Japan).

All animals were treated in accordance with the Ethical Guidelines for Investigations of Experimental Animals of the Nippon Dental University School of Life Dentistry at Niigata (No. 219).

## 5. Conclusions

NIR-PIT using the EGFR Affibody–IR700Dye conjugate induced necrotic cell death in EGFR-positive salivary gland cancer cells without causing any damage to the control cells. It also successfully slowed down the growth of EGFR-positive salivary gland tumors. Therefore, the use of Affibody may extend the therapeutic prospects of NIR-PIT for the treatment of EGFR-positive salivary gland cancer.

## Data Availability

Data is contained within the article; further inquiries can be directed to the corresponding author.
